# Magnetite Nanoparticles with High Affinity Toward Target Protein for Efficient and Facile Bio‐Separation

**DOI:** 10.1002/advs.202413605

**Published:** 2025-03-16

**Authors:** Yafei Wang, Yaojing Zhang, Yibo Zhao, Zhuo Zhao, Jia Yao, Lei Zou, Yan Zhang, Ying Guan, Yongjun Zhang

**Affiliations:** ^1^ State Key Laboratory of Separation Membranes and Membrane Processes School of Pharmaceutical Sciences Tiangong University Tianjin 300387 China; ^2^ Cangzhou Institute of Tiangong University Cangzhou 061000 China; ^3^ State Key Laboratory of Separation Membranes and Membrane Processes School of Chemistry Tiangong University Tianjin 300387 China; ^4^ Institute of Polymer Chemistry College of Chemistry Nankai University Tianjin 300071 China

**Keywords:** bio‐separation, magnetite nanoparticles, molecular imprinting, proteins

## Abstract

Magnetite nanoparticles (Fe_3_O_4_ NPs) with molecular recognition capabilities offer significant potential for biomedical applications, yet existing surface protein imprinting methods often suffer from low efficiency. Herein, a surface enzyme‐mediated polymerization strategy is exploited for surface imprinting of bovine serum albumin (BSA) onto Fe_3_O_4_ NPs. This method, compatible with all vinyl monomers and operable under mild conditions, enables imprinting at high monomer concentrations while preventing nanoparticle agglomeration. Notably, increasing the pre‐polymerization solution concentration enhances the pre‐assembly of functional monomers and template molecules, thereby improving imprinting efficiency. Furthermore, replacing conventional crosslinkers with a polyglutamic acid‐based peptide crosslinker introduces a pH‐responsive helix‐coil transition, allowing complete template removal under mild conditions and increasing the adsorption capacity and imprinting factor to 139.8 mg g⁻¹ and 10.36, respectively. The resulting BSA‐imprinted Fe₃O₄ NPs exhibits high selectivity, robustness, and rapid adsorption kinetics while maintaining strong magnetic responsiveness for easy separation. These features allows for the selective extraction of BSA from bovine fetal serum, demonstrating the potential of this approach for biomedical applications, particularly in bioseparations.

## Graphical abstract

BSA‐imprinted magnetite nanoparticles with high adsorption capacity, high selectivity, fast adsorption kinetics, and high magnetic susceptibility are synthesized using surface enzyme‐mediated polymerization and polyglutamic acid‐based peptide crosslinker.

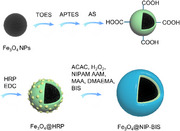

## 1 Introduction

Magnetite nanoparticles (Fe_3_O_4_ NPs) have been widely exploited as novel biomedical nanomaterials for drug delivery, hyperthermia, magnetic resonance imaging, bioseparation, etc., taking advantage of their low cytotoxicity and high saturation magnetization.^[^ ^1^, ^2^, ^3^, ^4^ ^]^ Surface modification of the particles plays a critical role in these applications. First of all, coating the particles with an inorganic or organic shell prevents the oxidation and aggregation of the particles, and thus enhances their colloidal stability and biocompatibility.^[^ ^5^ ^]^ More importantly, surface modification renders the particles capable of recognizing the target biomarkers, which is indispensable for their applications in bioseparation,^[^ ^6^ ^]^ cancer cell separation,^[^ ^7^ ^]^ drug delivery,^[^ ^8^ ^]^ and magnetic resonance imaging.^[^ ^9^, ^10^ ^]^

Modification with antibodies is a straightforward way to introduce recognition capability,^[^ ^7^, ^8^, ^9^, ^10^ ^]^ however, natural antibodies suffer from high cost and instability. A promising alternative is surface molecular imprinting, particularly surface protein imprinting, over Fe_3_O_4_ NPs.^[^ ^11^ ^]^ In molecular imprinting, specific recognition sites are created by polymerization using the target molecules as templates.^[^ ^12^, ^13^ ^]^ Unlike antibodies, molecularly imprinted polymers (MIPs), as synthetic receptors, are cheap and robust.^[^ ^12^ ^]^ Unfortunately, because of the large size and complex structure of proteins, imprinting of proteins still faces many challenges, including difficult template removal, slow binding kinetics, and low imprinting efficiency.^[^ ^14^, ^15^, ^16^, ^17^, ^18^, ^19^, ^20^, ^21^ ^]^ In surface protein imprinting, the template removal and binding kinetics problems are alleviated as the recognition sites are situated at or close to the surface of MIPs, however, the imprinting efficiency is still low.^[^ ^22^ ^]^

Herein to improve imprinting efficiency, for the first time, surface enzyme‐mediated polymerization^[^ ^23^, ^24^ ^]^ was exploited for surface protein imprinting over Fe_3_O_4_ NPs. Previously surface‐initiated polymerization methods, such as surface‐initiated photopolymerization^[^ ^25^ ^]^ or atomic transfer radical polymerization (ATRP)^[^ ^26^ ^]^ were used for surface protein imprinting. The agglomeration of nanoparticles, a severe problem in surface protein imprinting, is successfully avoided. However, these methods suffer from UV light‐induced conformational change of template protein (photopolymerization)^[^ ^27^ ^]^ or limited selection of monomers and potential toxicity of residual metals (ATRP).^[^ ^28^, ^29^ ^]^ To address these problems, the surface graft polymerization method was proposed by Fu et al.^[^ ^30^ ^]^ This method uses common redox initiators and is compatible with all vinyl monomers. However, to avoid the agglomeration of nanoparticles and possible gelation of the reaction dispersion, it can only be carried out at a low monomer concentration.^[^ ^28^, ^30^, ^31^ ^]^ Like the previously used surface‐initiated polymerization methods, surface enzyme‐mediated polymerization can avoid the agglomeration of nanoparticles, even at high monomer concentrations. At the same time, this method can be carried out under mild conditions and is compatible with all vinyl monomers. More importantly, this method can be performed at high monomer concentrations, leading to enhanced pre‐assembly between the template and functional monomers and hence improved imprinting efficiency. To further improve imprinting efficiency the conventional crosslinker was replaced with a polyglutamic acid‐based peptide crosslinker. Because the peptide segments are capable of undergoing reversible and precise pH‐induced helix‐coil transition, the resulting imprint cavities are shape‐memorable, leading to complete template removal under mild conditions and significantly improved imprinting efficiency.^[^ ^32^, ^33^, ^34^ ^]^ In this way surface BSA‐imprinted Fe_3_O_4_ NPs with high adsorption capacity (139.8 mg g^−1^) and high imprint factor (IF) (10.36) were successfully synthesized. The resulting hybrid nanomaterials, combining magnetic properties of Fe_3_O_4_ NPs with the high recognition capability of imprinted polymers, are expected to find important applications such as magnetic bioseparation.

## 2 Results and Discussion

**Figure 1:**
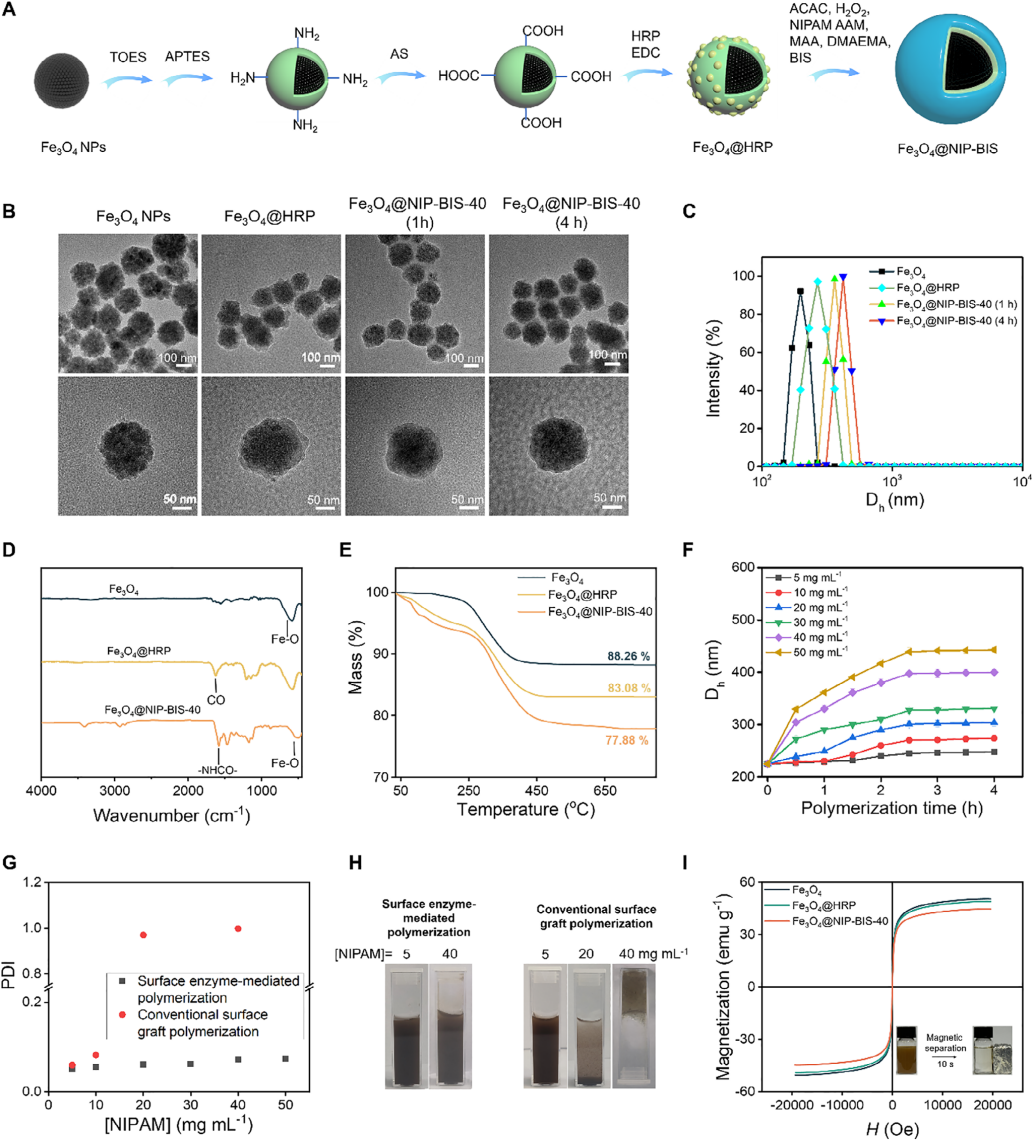
Synthesis of polymer shell over Fe_3_O_4_ NPs via surface enzyme‐mediated polymerization. A) Schematic illustration of the synthesis process. B) TEM images of Fe_3_O_4_ NPs, Fe_3_O_4_@HRP, and Fe_3_O_4_@NIP‐BIS‐40 were synthesized using a polymerization time of 1 and 4 h. C) Hydrodynamic diameter (*D_h_*) distribution of the nanoparticles. D,E) FTIR spectra (D) and thermogravimetric analysis (E) of the nanoparticles. F) Hydrodynamic diameter (*D_h_*) of Fe_3_O_4_@ NIP‐BIS nanoparticles synthesized using different monomer concentrations and polymerization time. G,H) PDI (G) and photographs (H) of Fe_3_O_4_@NIP‐BIS synthesized using two different polymerization methods and monomer concentrations. I) Magnetization curves of the nanoparticles.

To overcome the limitations of the polymerization methods used previously, herein surface enzyme‐mediated polymerization^[^ ^23^, ^24^ ^]^ was exploited to synthesize the protein‐imprinted polymer shells on Fe_3_O_4_ NPs (**Figure** **1**A). The Fe_3_O_4_ NPs were synthesized via a solvothermal method.^[^ ^35^ ^]^ To immobilize HRP onto the particle surface, first a silica shell was coated via the hydrolysis and condensation of TEOS. The addition of a silica shell not only allows further modification of Fe_3_O_4_ NPs but also improves their stability and dispersibility in various solvents.^[^ ^11^, ^36^ ^]^ The amino groups were then introduced via the hydrolysis‐condensation of APTES. These amino‐modified particles were further treated with succinic anhydride (SA) to yield carboxylic acid‐modified particles. Finally, HRP was anchored onto the surface of the particles using EDC/NHS as coupling agents. As TEM images reveal, the HRP‐modified particles, i.e., Fe_3_O_4_@HRP, remain to be monodisperse, just like the bare Fe_3_O_4_ NPs (Figure 1B). Dynamic light scattering (DLS) study confirms the monodispersity of the NPs, as both NPs exhibit a PDI lower than 0.1 (0.025 for Fe_3_O_4_ and 0.050 for Fe_3_O_4_@HRP). DLS data also reveal that the hydrodynamic diameter (*D_h_*) of the nanoparticles increases from 207 to 225 nm after HRP immobilization (Figure 1C). The successful immobilization of HRP was further verified by the color change of H_2_O_2_/3,3′,5,5′‐tetramethylbenzidine (TMB) mixed solution from colorless to brown–yellow upon addition of Fe_3_O_4_@HRP NPs (Figure S1A,B, Supporting Information). In addition, an adsorption peak was observed at 450 nm on the UV–vis spectra of the solution, indicating the formation of oxidized TMB product (Figure S1C,D, Supporting Information). These results not only confirm the successful immobilization of HRP on the particles but also indicate the immobilized enzyme retains its catalytic activity.

To check if the immobilized enzyme can mediate polymerization and fabricate a polymer shell over the NPs, Fe_3_O_4_@HRP NPs were added into pre‐polymerization solutions containing both monomers and crosslinker. H_2_O_2_ and acetylacetone (ACAC) were then added to initiate the polymerization. A series of pre‐polymerization solutions were prepared in which the molar ratio of the monomers and cross‐linker BIS was kept constant while the total concentration varied, so the concentration of the solution was represented by the concentration of NIPAM, [NIPAM] (Table S1, Supporting Information). The resulting particles were actually the non‐imprinted particles for the surface BSA‐imprinted particles. Therefore, they were named Fe_3_O_4_@NIP‐BIS‐x, where x indicated [NIPAM] in the pre‐polymerization solution. As shown in Figure 1C, after 1 h of polymerization in [NIPAM] = 40 mg mL^−1^ solution, *D_h_* of the particles increases from 225 to 331 nm, which further increases to 400 nm after 4 h of polymerization. Meanwhile, the PDI value only slightly increases from 0.050 to 0.072 after 4 h of polymerization, indicating good monodispersity of the particles. The monodispersity of the particles was also confirmed by TEM examination (Figure 1B). The significantly increased size of the particles indicates the successful addition of a polymer shell on the particles. It has long known that HRP can medicate the free‐radical polymerization of various vinyl monomers.^[^ ^23^, ^24^, ^37^, ^38^ ^]^ As shown in Scheme S1 (Supporting Information), HRP catalyzes the oxidation of ACAC by H_2_O_2_, generating ACAC radicals which initiate the polymerization of vinyl monomers.^[^ ^38^, ^39^, ^40^ ^]^ Herein because of the presence of crosslinker BIS, the HRP‐medicated polymerization produces a thin hydrogel shell around the particles. The successful synthesis of the polymer shell was also confirmed by the appearance of new bands assigned to amide groups (1625 and 1534 cm^−1^) and isopropyl groups (1386 and 1369 cm^−1^) in FTIR spectra of the particles (Figure 1D) and increased mass loss in thermogravimetric analysis (Figure 1E).

The effects of polymerization time and monomer concentration were studied. As shown in Figure 1F, the particle size increases with increasing polymerization time and levels off when polymerization time exceeds 2.5 h. When keeping the polymerization time constant, a higher monomer concentration results in a thicker polymer shell. These results suggest the polymer shell thickness can be controlled by altering the monomer concentration and/or polymerization time.

Unlike the previously used surface‐initiated polymerization methods, surface enzyme‐mediated polymerization can be performed under mild conditions and is compatible with almost all vinyl monomers.^[^ ^24^ ^]^ Meanwhile, it can be carried out at a high monomer concentration. In contrast, although the conventional surface graft polymerization method is compatible with all vinyl monomers, it can only perform at a low monomer concentration to avoid particle agglomeration.^[^ ^30^ ^]^ As shown in Figure 1G, the PDI of the particles synthesized by surface enzyme‐mediated polymerization remains very low, even for those synthesized at high monomer concentrations, indicating excellent monodispersity of these particles. In this method, the radicals are generated at the particle‐solution interface, therefore the polymerization can only occur in the close vicinity of the particle surface, thus avoiding the agglomeration of the particles during polymerization. These particles can be well‐dispersed in water, and the resulting dispersions are colloidally stable (Figure 1H). We also used the conventional surface graft polymerization to synthesize a polymer shell over vinyl‐modified Fe_3_O_4_ NPs, in which the polymerization was initiated by adding APS and TEMED. The same pre‐polymerization solutions were used. As shown in Figure 1G, only at low monomer concentrations, e.g., [NIPAM] = 5 and 10 mg mL^−1^, this method can produce relatively monodisperse particles. As monomer concentration increases, the PDI of the resulting particles increases dramatically, suggesting severe agglomeration of the particles. Increasing monomer concentration to [NIPAM] = 40 mg mL^−1^ even leads to the gelation of the solution (Figure 1H). In conventional surface graft polymerization, the radicals are generated everywhere in the solution. Therefore, the aggregation of NPs is inevitable at a high monomer concentration. These results can explain why a very low monomer concentration was used in previous efforts using conventional surface graft polymerization.^[^ ^30^ ^]^

Magnetic responsibility is the most important property of Fe_3_O_4_ NPs. VSM analysis reveals that the magnetic saturation (Ms) value of the unmodified Fe_3_O_4_ NP is ≈50.5 emu g^−1^ (Figure 1I). The value is reduced to be ≈49.1 emu g^−1^ after modified with HRP and further reduced to be ≈44.6 emu g^−1^ after coated with a polymer shell. The reduced Ms values are attributed to the reduced contents of magnetite in the modified particles.^[^ ^27^, ^31^, ^41^ ^]^ Although the Ms values are reduced slightly, the magnetic susceptibility of the modified particles is still high enough, therefore they can be separated facilely by applying an external magnetic field, as shown in the inset of Figure 1I.

**Figure 2:**
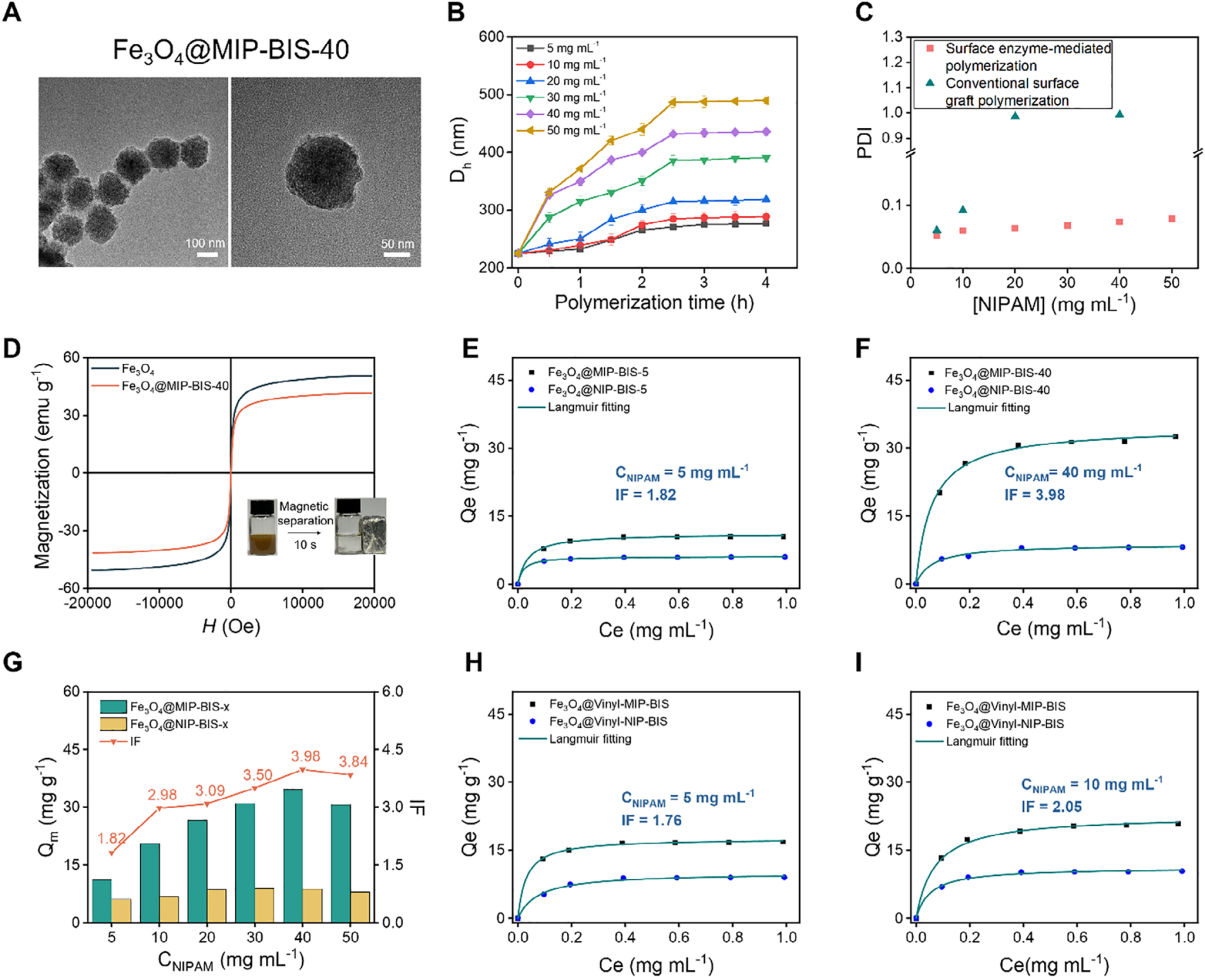
Synthesis of BIS‐crosslinked BSA‐imprinted magnetite nanoparticles via surface enzyme‐mediated polymerization. A) TEM images of Fe_3_O_4_@MIP‐BIS‐40 nanoparticles. B) *D_h_* of the particles synthesized using different [NIPAM] and polymerization times. C) PDI of the BSA‐imprinted nanoparticles synthesized via two different polymerization methods. D) Magnetization curves of Fe_3_O_4_ and Fe_3_O_4_@MIP‐BIS‐40; E,F) Adsorption isotherms of BSA on BSA‐imprinted and corresponding non‐imprinted particles synthesized at [NIPAM] = 5 mg mL^−1^ (E) and 40 mg mL^−1^ (F). G) Q_m_ and IF value of Fe_3_O_4_@MIP‐BIS‐x synthesized at different [NIPAM]. H,I) Adsorption isotherms of BSA on BSA‐imprinted and corresponding non‐imprinted particles synthesized via traditional graft polymerization at [NIPAM] = 5 mg mL^−1^ (H) and 10 mg mL^−1^(I).

After demonstrating that surface enzyme‐mediated polymerization allows the fabrication of polymer nanocoatings on Fe_3_O_4_ NPs at high monomer concentrations, it was further exploited for surface protein imprinting. For this purpose, the template protein BSA was added to the pre‐polymerization solutions (Table S1, Supporting Information). TEM examination reveals the resulting BSA‐imprinted particles, named Fe_3_O_4_@MIP‐BIS‐40, are also monodisperse and exhibit a similar morphology to the non‐imprinted particles (**Figure** **2**A). Again, the appearance of new bands in FTIR spectra (Figure S2, Supporting Information) and increased mass loss in TGA (Figure S3, Supporting Information) confirm the successful synthesis of the polymer nanocoating. Note the mass loss of Fe_3_O_4_@MIP‐BIS‐40 (6.0% compared to Fe_3_O_4_@HRP) is larger than that of the corresponding Fe_3_O_4_@NIP‐BIS‐40 (5.2% compared to Fe_3_O_4_@HRP) (Figure 1E), suggesting a thicker coating was added in the presence of the template protein.

Similar to the non‐imprinted particles, the D_h_ of BSA‐imprinted particles increases with polymerization time and levels off after 2.5 h of polymerization (Figure 2B). It also increases with increasing monomer concentration when keeping the polymerization time constant. From the D_h_ of the particles, the thickness of the polymer coating was calculated. As shown in Figure S4 (Supporting Information), the thickness of the polymer coating increases almost linearly with increasing monomer concentration. Again, it was found that the imprinted layer is thicker than the non‐imprinted layer, which is in agreement with the TGA results (Figure S3, Supporting Information). Like the non‐imprinted particles, the PDI of the imprinted particles remains below 0.1, indicating excellent monodispersity (Figure 2C). In contrast, when synthesizing BSA‐imprinted particles via the conventional surface graft polymerization, severe aggregation occurs when using a high monomer concentration, as indicated by a PDI of ≈0.9 at [NIPAM] = 20 mg mL^−1^ (Figure 2C). The BSA‐imprinted particles maintain a high magnetic susceptibility. The Ms value was determined to be 41.5 emu g^−1^ for Fe_3_O_4_@MIP‐BIS‐40, allowing it to be separated from the solution easily using a magnet (Figure 2D).

After removing the template protein by eluting it in 0.5 m NaCl, the rebinding of BSA by the imprinted particles was examined. The adsorption kinetics of BSA onto the particles were first studied (Figure S5, Supporting Information). For Fe_3_O_4_@MIP‐BIS‐40, adsorption equilibrium was reached within ≈40 min. The rapid adsorption kinetics observed for Fe_3_O_4_@MIP‐BIS‐40 are consistent with previous studies on surface‐imprinted particles.^[^ ^42^ ^]^ Figure 2E shows the adsorption isotherms of the particles synthesized at [NIPAM] = 5 mg mL^−1^. The isotherm can be well‐described by the Langmuir model.^[^ ^32^ ^]^ From Langmuir fitting, the maximum adsorption capacity (Q_m_) was determined to be 11.12 and 6.13 mg g^−1^ for the imprinted and non‐imprinted particles, respectively. Imprinting factor (IF), the ratio of Q_m_ of the imprinted particle to the non‐imprinted one, was thus determined to be 1.82. The larger Q_m_ of the imprinted particle than the corresponding non‐imprinted one indicates specific binding sites are successfully created by molecular imprinting.

The Q_m_ and IF values of other particles were also determined in the same way. As an example, Figure 2F shows the adsorption isotherms of the particles synthesized at [NIPAM] = 40 mg mL^−1^. It is interesting to find that both Q_m_ and IF increase with increasing monomer concentration until [NIPAM] reaches 40 mg mL^−1^ (Figure 2G). Particularly, when [NIPAM] increases to 40 mg mL^−1^, Q_m_ increases to 35 mg g^−1^, and IF increases to 3.98 (Figure 2F). The significant improvement in imprinting efficiency can be attributed to the more efficient pre‐assembly of functional monomers and the template protein at higher monomer concentrations. In molecular imprinting, the functional monomers first assemble around the template via various interactions, e.g., electrostatic interaction, hydrophobic interaction, and hydrogen bonding. The positions of the functional groups are then fixed by polymerization of the monomers. Therefore, the removal of the template will leave imprint cavities complementary to the template in size, shape, and chemical functionalities.^[^ ^12^ ^]^ One can see pre‐assembly between the functional monomers and the template plays a critical role in molecular imprinting. Since the preassembly is usually achieved via dynamic interactions, it will not be efficient at a low monomer concentration, thus leading to a low imprinting efficiency as we observed for Fe_3_O_4_@MIP‐BIS‐5 (Figure 2E). As monomer concentration increases, however, the pre‐assembly between the functional monomers and the template will be enhanced, thus leading to improved imprinting efficiency.

Surface imprinting of BSA over Fe_3_O_4_ NPs was also carried out using the same pre‐polymerization solutions but the conventional surface graft polymerization, i.e., initiated by adding APS and TEMED. A slightly increased imprinting efficiency was also observed when [NIPAM] increased from 5 to 10 mg mL^−1^ (Q_m_ increases from 18 to 23 mg g^−1^, while IF increases from 1.76 to 2.05) (Figure 2H,I). Therefore, these results further demonstrated that the pre‐assembly of functional monomers and the template protein at higher monomer concentrations can enhance imprinting efficiency. Unfortunately, further improving imprinting efficiency by increasing monomer concentration failed because of the severe agglomeration of the particles (Figure 2C).

**Figure 3:**
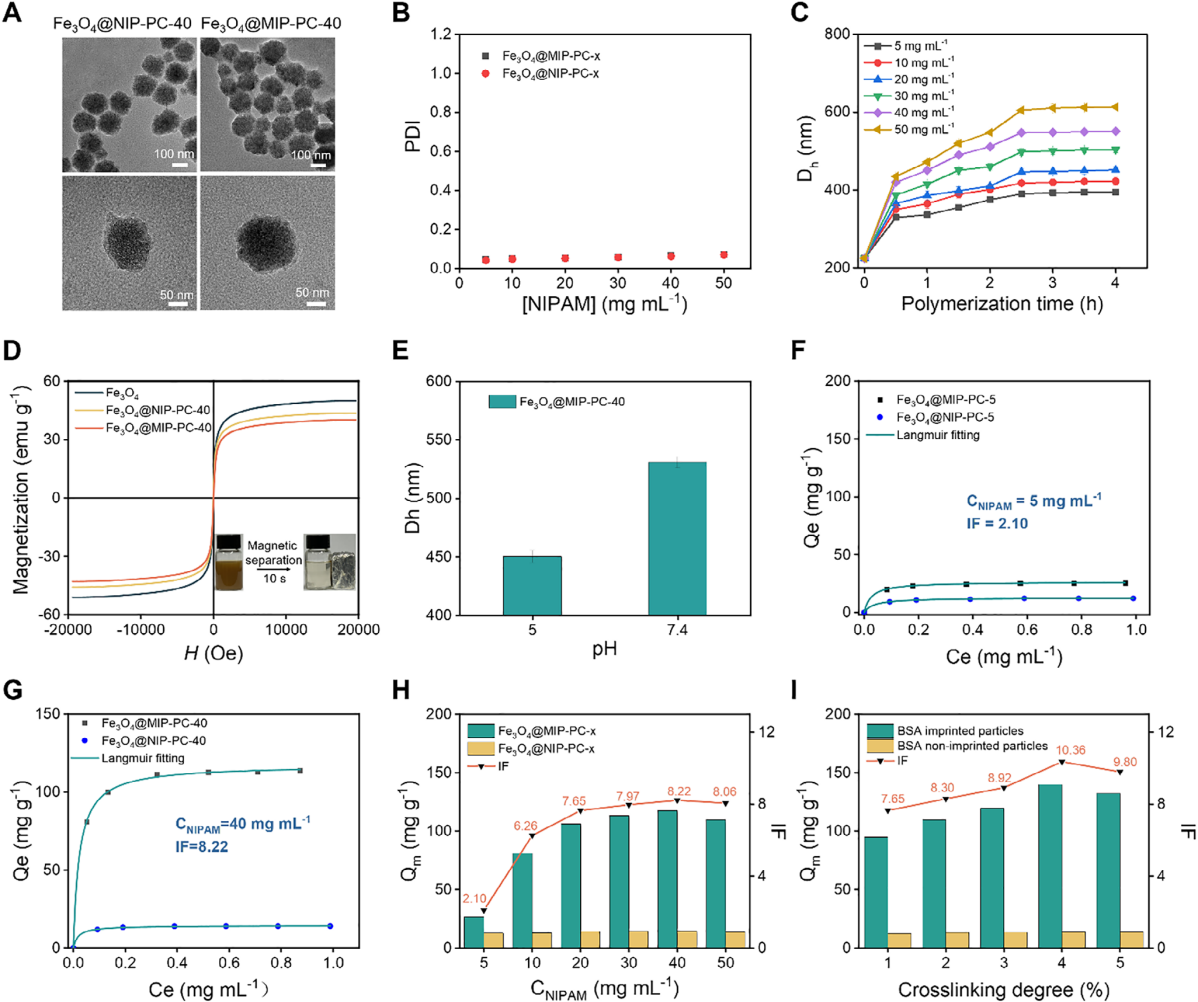
Synthesis of PC‐crosslinked BSA‐imprinted magnetite nanoparticles via surface enzyme‐mediated polymerization. A) TEM images of Fe_3_O_4_@MIP‐PC‐40 and Fe_3_O_4_@NIP‐PC‐40. B) PDI of the particles synthesized at different [NIPAM]. C) *D_h_* of the particles synthesized using different [NIPAM] and polymerization times. D) Magnetization curves of the particles. E) *D_h_* of Fe_3_O_4_@MIP‐PC‐40 at different pHs. F,G) Adsorption isotherms of BSA on the BSA‐imprinted and corresponding non‐imprinted particles synthesized at [NIPAM] = 5 mg mL^−1^ (F) and 40 mg mL^−1^ (G). H) Q_m_ and IF of the BSA‐imprinted particles synthesized at different [NIPAM]. I) Q_m_ and IF of the BSA‐imprinted particles synthesized using different crosslinking degrees.

We previously demonstrated that the two major challenges in bulk protein imprinting, i.e., the difficult template removal and low imprinting efficiency, can be overcome by replacing the conventional crosslinker with peptide crosslinkers.^[^ ^32^, ^33^, ^34^ ^]^ This strategy was adopted to further improve the imprinting efficiency of the BSA‐imprinted Fe_3_O_4_ NPs. For this purpose, a polyglutamic acid‐based peptide crosslinker (PC) was synthesized (Figure S6A, Supporting Information). The polymerization degree of the PC was determined to be 21. Like other polyglutamic acids, the PC undergoes pH‐induced helix‐coil transition. It adopts an α‐helical conformation at pH 5.0, but a coiled conformation at pH 7.4 (Figure S6B, Supporting Information). The same recipe of the pre‐polymerization solution was used expecting that the crosslinker BIS was substituted with the equimolar of PC (Table S2, Supporting Information). Note the pH of the pre‐polymerization solution was adjusted to 5.0 to allow the PC to adopt α‐helical conformation when polymerization. The resulting PC‐crosslinked BSA‐imprinted particles were named Fe_3_O_4_@MIP‐PC‐x, and the corresponding non‐imprinted particles, Fe_3_O_4_@NIP‐PC‐x, where x indicates [NIPAM] in the pre‐polymerization solution. The successful synthesis of these particles was again demonstrated by TEM, FTIR, and TGA studies (**Figure** **3**A; Figure S7, Supporting Information). Like the BIS‐crosslinked particles, the PC‐crosslinked particles are also monodisperse (Figure 3A,B). The shell thickness of the PC‐crosslinked particles can also be controlled by monomer concentration and polymerization time (Figure 3C; Figure S8, Supporting Information). Because of their high magnetic susceptibility, they can also be facilely separated using a magnet (Figure 3D). XRD study also shows that the crystal structure of the Fe_3_O_4_ core was essentially maintained after the surface enzyme‐mediated polymerization and imprinting process (Figure S9, Supporting Information).

Unlike the BIS‐crosslinked particles, for the PC‐crosslinked particles, the BSA template can be facilely eluted using a pH 7.4 buffer. Adjusting pH from 5.0 to 7.4 renders the polyglutamic acid segments in the polymer shell to convert from α‐helical conformation to coiled conformation, resulting in dramatic swelling of the polymer shell, as evidenced by the dramatic increase in the *D_h_* of particles (Figure 3E). The pH‐induced swelling of the polymer shell facilitates the elution of the protein template. As a result, almost complete template removal could be achieved under mild conditions.^[^ ^32^, ^33^ ^]^

The rebinding of BSA was then carried out in a pH 5.0 buffer. Again, improved imprinting efficiency was observed when increasing the monomer concentration of the pre‐polymerization solution (Figure 3F–H). At [NIPAM] = 5 mg mL^−1^, the Q_m_ was determined to be 26.5 mg g^−1^ and IF was determined to be 2.10 (Figure 3F). Increasing [NIPAM] to 40 mg mL^−1^ significantly increases Q_m_ to 117.5 mg g^−1^ and IF to 8.22 (Figure 3G). These results should also be attributed to the more efficient pre‐assembly at a higher monomer concentration. In addition, both Q_m_ and IF are significantly higher than that of the BIS‐crosslinked particles synthesized at the same [NIPAM], which is 35 mg g^−1^ and 3.98, respectively (Figure 2F). When pH was adjusted back to 5.0, the polyglutamic acid segments in the polymer shell folded back into α‐helix. Because of the precise folding of the peptide segments, not only the size but also the shape of the imprint cavities will be restored. Therefore, the PC‐crosslinked particles demonstrate a much higher imprinting efficiency than the BIS‐crosslinked particles.^[^ ^32^, ^33^ ^]^

A constant crosslinking degree of 1%, i.e., molar ratio of crosslinker to total monomers, was used in the above studies. Considering the key role of PC, the influence of PC content on imprinting efficiency was further investigated. As shown in Figure 3I, both Q_m_ and IF increase when increasing the crosslinking degree from 1% to 4%, but fall when further increasing the crosslinking degree to 5%. At the optimal cross‐linking degree, i.e., 4%, Q_m_ reaches 139.8 mg g^−1^, and IF reaches 10.36. **Table** **1** compares the performance of surface protein‐imprinted Fe_3_O_4_ NPs reported in the literature. The BSA‐imprinted particles synthesized here present the highest imprinting efficiency, in terms of both specific binding (i.e., Q_m,MIP_ – Q_m,NIP_) and IF value. In addition, the template protein is removed under mild conditions, while harsh conditions, for example, elution with acetic acid/SDS^[^ ^27^ ^]^ or high concentration NaCl,^[^ ^28^ ^]^ were used in previous efforts (Table 1).

**Table 1:** Comparison of Fe_3_O_4_@MIP‐PC‐4% with surface protein‐imprinted Fe_3_O_4_ NPs reported in the literature.

Polymerization Method	Template	[Monomer]	Template Removal	Q_m,MIP_ [mg g^−1^]	Specific Binding [mg g^−1^]	IF	Refs.
Surface enzyme‐medicated polymerization	BSA	4.3%	pH 7.4 phosphate buffer	139.8	126.3	10.36	This work
Surface‐initiated ATRP	Lyz	1.38%	10%(v/v) acetic acid–10% (w/v) SDS	8.3	4.19	2.02	[27]
Surface‐initiated ATRP	lysozyme	4.08 wt%	Acetic acid (2%, v/v) and SDS (2%, w/v) 1: 1	243.65	56.2	1.30	[43]
Surface‐initiated ATRP	BSA	1.38%	10% (v/v) acetic acid–10% (w/v) SDS	11.62	5.87	2.02	[26]
Surface photopolymerization	green fluorescent protein (GFP), BSA, OVA, Lyz	0.445 wt.%	9/1 methanol/acetic acid	183(BSA)	130	3.33	[25]
Surface polymerization via iniferter	BSA	1.73 wt.%	9/1 methanol/acetic acid	294	214	3.68	[44]
surface graft polymerization using low monomer concentration	Lyz	0.40%	0.5 m NaCl	17.1	11.3	2.95	[31]
surface graft polymerization using low monomer concentration	lyz	0.4%	0.5 m NaCl	19.54	6.93	1.55	[28]
surface graft polymerization using low monomer concentration over silica nanoparticles	lyz	0.40%	0.5 m NaCl	11.3	2.11	1.23	[30]
surface graft polymerization using low monomer concentration	BSA	0.6%	SDS (10%, w/v) and acetic acid (10%, v/v)	78	31.8	1.69	[42]
surface graft polymerization using low monomer concentration	Lysozyme	0.5%	10% (W/V) SDS‐10% (V/V) HAc	166	103.8	2.67	[45]
surface graft polymerization using low monomer concentration	BHb	0.1%	10% (v/v) acetic acid containing 10% (w/v) SDS	77.6	52.6	3.1‐4.3	[46]

**Figure 4:**
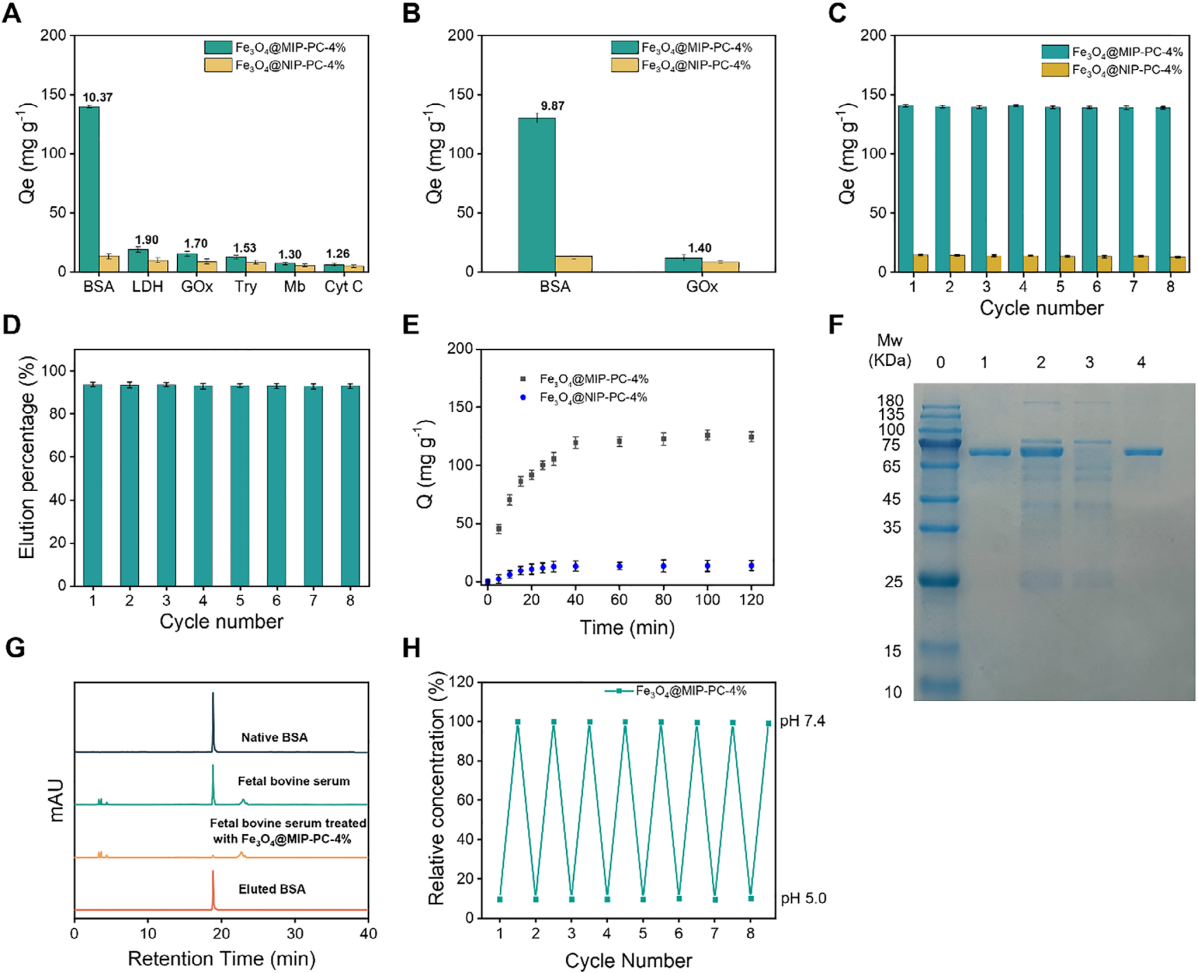
Characterization and applications of Fe_3_O_4_@MIP‐PC‐4%. A) Adsorption of different proteins onto the BSA‐imprinted and non‐imprinted particles. (C_0_ = 0.5 mg mL^−1^, pH = 5.0). B) Adsorption of BSA and GOx onto the BSA‐imprinted and non‐imprinted particles from a mixed solution of BSA and GOx. (C_0,BSA_ = C_0,GOx_ = 0.25 mg mL^−1^, pH = 5.0). C,D) Repeated adsorption of BSA onto the particles. C: Adsorption amount. (C_0_ = 0.5 mg mL^−1^, pH = 5.0). D: Elution percentage. (pH = 7.4). E) Adsorption kinetics of BSA onto the particles. (C_0_ = 0.5 mg mL^−1^, pH = 5.0). F,G) Separation of BSA from fetal bovine serum using Fe_3_O_4_@MIP‐PC‐4%. F: SDS‐PAGE analysis. Lane 0: protein marker. Lane 1: commercial BSA. Lane 2: 50‐fold diluted fetal bovine serum. Lane 3: fetal bovine serum after adsorption. Lane 4: Eluate from Fe_3_O_4_@MIP‐PC‐4%. G: HPLC analysis. H) Concentration changes of a BSA solution upon repeated pH changes between 5.0 and 7.4 in the presence of Fe_3_O_4_@MIP‐PC‐4%.

The PC‐crosslinked BSA‐imprinted and corresponding non‐imprinted particles with the optimal cross‐linking degree were named Fe_3_O_4_@MIP‐PC‐4% and Fe_3_O_4_@NIP‐PC‐4% and subjected to further studies. As shown in **Figure** **4**A, Fe_3_O_4_@MIP‐PC‐4% exhibits a high selectivity toward the template BSA. It adsorbs much more BSA, the template, than the non‐template proteins such as LDH, GOx, Try, Mb, and Cyt C, from their solutions in a pH 5.0 buffer. The IF for BSA is significantly higher than that for the non‐template proteins. Fe_3_O_4_@MIP‐PC‐4% was also used to adsorb proteins from a mixed solution containing an equal amount of BSA and GOx. As shown in Figure 4B, much more BSA was adsorbed than GOx, indicating the BSA‐imprinted particle retains its high affinity toward BSA even under the competition of another protein.

The PC‐crosslinked particles are highly robust. As shown in Figure 4C,D, the particles can be repeatedly used to adsorb BSA from a pH 5.0 solution and then eluted in a pH 7.4 buffer. In each cycle, almost the same amount of BSA was adsorbed, and the elution percentage remained as high as 93%. Almost no change in adsorption capacity and elution efficiency was found after 8 absorption‐elution cycles (Figure 4C,D). Like other surface‐imprinted particles, Fe_3_O_4_@MIP‐PC‐4% also features rapid adsorption kinetics. The adsorption of BSA reaches equilibrium within 40 min (Figure 4E). In contrast, for the PC‐crosslinked bulky imprinted polymers, it typically takes 8–12 h to reach adsorption equilibrium.^[^ ^47^, ^48^, ^49^, ^50^, ^51^, ^52^, ^53^ ^]^ The fast adsorption kinetics is attributed to the relatively thin surface‐imprinted coating, which makes the imprint cavities easily accessed by the protein molecules.

The combination of magnetic properties of Fe_3_O_4_ NP and high affinity toward the target protein makes Fe_3_O_4_@MIP‐PC‐4% potential for many innovative applications, such as the capture and separation of BSA from complex biofluids.^[^ ^11^ ^]^ As a demonstration, it was used to extract BSA from bovine fetal serum. The pristine serum sample contains numerous proteins, including BSA, as revealed by SDS‐PAGE analysis (Lane 2 in Figure 4F). After being treated with Fe_3_O_4_@MIP‐PC‐4%, the BSA band disappeared almost completely, while little change was found for other bands, demonstrating BSA was selectively removed from the sample (Lane 3 in Figure 4F). The particles were then eluted using a pH 7.4 buffer, and the eluate contained almost exclusively BSA (Lane 4 in Figure 4F). HPLC analysis (Figure 4G) also demonstrated that after treatment with Fe_3_O_4_@MIP‐PC‐4%, the BSA peak almost completely disappeared, and the eluate displayed only the peak of BSA. The extraction ratio was calculated to be ≈90% and the purity of the eluted BSA exceeds 95%. Thanks to the fast adsorption kinetics of the particles, it takes a relatively short time (≈40 min) for the adsorption step. More importantly, taking advantage of the magnetic properties of the particles, the particles can be facilely separated from the solution using a magnet.

The pH‐dependent BSA‐affinity of Fe_3_O_4_@MIP‐PC‐4% also makes it potential for on‐demand capture and release of BSA. As shown in Figure 4H, when Fe_3_O_4_@MIP‐PC‐4% was added into a BSA solution at pH 5.0, the BSA concentration dropped sharply to ≈10% of its original value, indicating ≈90% BSA in the solution was captured by the particles. Upon adjusting pH to 7.4 by adding 1 m NaOH, the BSA concentration in the solution was restored to over 99% of its original value, indicating a nearly complete release of BSA from the particles into the solution. When pH was adjusted back to 5.0 by adding 1 m HCl, the BSA concentration dropped again to ≈10% of its original value. The BSA capture and release cycle was repeated 8 times. In each cycle, BSA concentration dropped to ≈10% at pH 5.0 and returned back to over 99%, demonstrating the high robustness of the particles.

## 3 Conclusion

In summary, for the first time, a novel initiation system composed of immobilized HRP, ACAC, and H_2_O_2_ was used in surface protein imprinting over Fe_3_O_4_ nanoparticles. Unlike the previously used surface‐initiated ATRP method, the surface enzyme‐mediated polymerization is compatible with all vinyl monomers. Meanwhile, unlike the conventional surface graft polymerization method, it can be performed at high monomer concentrations and avoid the agglomeration of the nanoparticles. More importantly, it was discovered that the imprinting efficiency increases with increasing concentration of the prepolymerization solution, which is attributed to the enhanced pre‐assembly between the functional monomers and the template protein. When the commonly‐used crosslinker N,N′‐methylenebis(acrylamide) was replaced with a polyglutamic acid‐based peptide crosslinker, we again found that the imprinting efficiency increases with increasing concentration of the prepolymerization solution. In addition, because the peptide segments could undergo pH‐induced helix‐coil transition, the imprint cavities are shaped memorable. Not only the template protein can be removed under mild conditions, the imprinting efficiency is further improved. Finally, BSA‐imprinted Fe_3_O_4_ NPs with high adsorption capacity and high imprinting factor were successfully synthesized. The new BSA‐imprinted Fe_3_O_4_ NPs exhibit high selectivity, high robustness, fast adsorption kinetics, and high magnetic susceptibility. When used to extract BSA selectively from bovine fetal serum, not only BSA can be selectively and efficiently separated from the complex biological sample, but the process is simple and fast, thanks to the combination of the high magnetic susceptibility of the Fe_3_O_4_ core and the high affinity of the imprinted layer toward BSA.

## Conflict of Interest

The authors declare no conflict of interest.

## Supporting information



Supporting Information

## Data Availability

The data that support the findings of this study are available from the corresponding author upon reasonable request.
