# Supplementary material for: Magnetite Nanoparticles with High Affinity Toward Target Protein for Efficient and Facile Bio‐Separation
Source: Adv Sci (Weinh). 2025 Mar 16;12(18):2413605. doi: 10.1002/advs.202413605 (PMC12079544; doi:10.1002/advs.202413605)
Supplement: Supplementary file 1 — Supporting Information [file ADVS-12-2413605-s001.docx]

Supporting Information

Magnetite Nanoparticles with High Affinity Towards Target Protein for Efficient and Facile Bio-separation

Yafei Wang, Yaojing Zhang, Yibo Zhao, Zhuo Zhao, Jia Yao, Lei Zou, Yan Zhang,* Ying Guan,* Yongjun Zhang*

Experimental section

*Materials*: 1-Ethyl-3-(3-dimethylaminopropyl)carbodiimide hydrochloride (EDC, 97%), triphosgene (99%), γ-benzyl-L-glutamate (BLG, 98%), 1-hydroxybenzotriazole (HOBT, 99%), acrylic acid (AA, 98%), poly(4-styrenesulfonic acid-co-maleic acid) sodium salt (average Mw~20000, ratio 3:1), sodium acetate anhydrous (99%), anisolewere (99%), succinic anhydride (99%) and N-hydroxysuccinimide (NHS, 98%) were purchased from Aladdin (Shanghai, China). Ferric chloride hexahydrate (FeCl_3_·6H_2_O, 98%) and acetylacetone (ACAC, 99.5%) were purchased from Macklin (Shanghai, China). Tetraethyl orthosilicate (TEOS, 99%), ethylene glycol (99.5%), trifluoroacetic acid (TFA, 99.9%), methanesulfonic acid (99%), methacrylic acid 3-trimethoxysilylpropyl ester (MPS，98%), 3-aminopropyltriethoxysilane (APTES, 98%), N,N′-methylenebis(acrylamide) (BIS, 98%), N-isopropylacrylamide (NIPAM, 98%), acrylamide (AAm, 98%), methacrylic acid (MAA, 99%), N, N, N′, N′-tetramethylenebis(acrylamide) (TEMED, 99%), dimethylaminoethyl methacrylate (DMAEMA, 99%), 3,3',5,5'-tetramethylbenzidine (TMB) and ammonium persulfate (APS, 98%) were purchased from J&K Scientific (Beijing, China). Ethyl acetate, n-hexane, methanol (MeOH), dichloromethane (DCM), hydrogen peroxide (98%), and diethyl ether were purchased from Windboat Chemical Reagent (Tianjin, China). Bovine serum albumin (BSA, Mw = 66.0 kDa, pI = 4.7) was purchased from Solarbio (Beijing, China). Horseradish peroxidase (HRP, Mw = 40.0 kDa, pI = 7.0) and cytochrome c (Cyt c, Mw = 12.5 kDa, pI = 10.8) were obtained from Yuanye (Shanghai, China). Trypsin (Try, Mw = 14.1 kDa, pI = 4.2), L-lactic dehydrogenase (LDH, Mw = 45 kDa, pI = 4.5), glucose oxidase (GOx, Mw = 160 kDa, pI = 4.9) and myoglobin (Mb, Mw = 16.7 kDa, pI = 7.07) were purchased from Sigma-Aldrich (Shanghai, Beijing). NIPAM was recrystallized in n-hexane/acetone (v/v = 10:1). MAA and DMAEMA were distilled under reduced pressure. Other regents were used without further treatment. The polyglutamic acid-based peptide crosslinker (PC) was synthesized using a procedure reported previously.*[1, 2]*

*Synthesis of PC: L-Glutamic acid 5-tert-butyl ester (10.0 g, 49.20 mmol) and triphosgene (6.3 g, 22.05 mmol) were dissolved in dried THF (150 mL) with magnetic stirring in a nitrogen atmosphere. After 2.5 hours of reaction at 50°C, the solution was precipitated into heptane with vigorous stirring. The solid product (Glu(t-Bu)-NCA) was collected by filtration and purified by recrystallization. Glu(t-Bu)-NCA (20.0 g, 87.25 mmol) was dissolved in dried DCM (400 mL) at room temperature, and then 3-buten-1-amine (399.0 μL, 4.36 mmol) was added to initiate the polymerization in a nitrogen atmosphere. After 48 hours of reaction, acrylic acid (1.5 mL, 21.80 mmol), EDC (4.1 g, 21.80 mmol), and HOBt (589.2 mg, 4.36 mmol) were added to the solution at 0°C to couple the amino end with the vinyl group. Subsequently, TFA (15 mL) was added to the mixture at 0°C to remove the tert-butyl protective group. The resulting product was purified by precipitation into diethyl ether, filtration, dialysis, and lyophilization.*

*Synthesis and modification of Fe_3_O_4_ NPs*: Fe_3_O_4_ NPs were synthesized by solvothermal method according to Ref*[3]*. To immobilize HRP onto Fe_3_O_4_ nanoparticles, the particles were first modified with amino groups. For this purpose, Fe_3_O_4_ NPs were added to a mixed solution of ethanol (400 mL), ultrapure water (12 mL) and ammonia (12 mL). After adding TEOS (0.1 mL), the mixture was stirred at 25°C for 2 h, and then APTES (12 mL) was added. The reaction was allowed to perform at 25°C for 24 hours. The product was separated using a magnet, washed three times with ethanol and ultrapure water, and then dried. The amino-modified particles were then dispersed in tetrahydrofuran (THF) (400 mL), followed by adding succinic anhydride (43.0 g). After stirring for 18 hours at 25°C, the particles were separated using a magnet, washed three times with ethanol and ultrapure water, and dried. The resulting carboxylic acid-modified particles were dispersed in 20 mM pH 5.8 phosphate buffer (100 mL). After adding EDC (0.40 g) and HRP (0.10 g), the solution was stirred for 4 hours. The resulting HRP-functionalized particles, Fe_3_O_4_@HRP, were separated using a magnet, washed three times with water, and dried.

The vinyl-modified Fe_3_O_4_ NPs were synthesized using a procedure similar to that for amino-modified particles, except that APTES was substituted with equivalent amount of MPS.

*Surface protein-imprinting over Fe_3_O_4_ NPs*: In a typical example, the template BSA (40.00 mg), AAm (45.22 mg), MAA (40.45 μL), DMAEMA (80.41 μL), NIPAM (2.00 g), BIS (29.68 mg) or peptide crosslinker (545.76 mg, 1% crosslinking degree), Fe_3_O_4_@HRP nanoparticles (250.00 mg) were sequentially added to 20 mM pH 5.0 phosphate buffer (50 mL). After purging with N_2_ for 15 min, ACAC (2.04 mL) and H_2_O_2_ (0.75 mL) were added to initiate polymerization. After 4 hours stirring at 25°C, the nanoparticles were separated using a magnet and washed three times with water to remove any unreacted monomer. To remove the template, the BIS-crosslinked particles were eluted with 0.5 M NaCl solution at 25°C, while the PC-crosslinked particles were eluted with phosphate buffer (pH 7.4). The non-imprinted particles were synthesized following the same procedure but in the absence of template protein.

Surface protein imprinting was also carried out by surface graft polymerization method*[4]*. As an example, the template BSA (40.00 mg), AAm (5.70 mg), MAA (5.10 μL), DMAEMA (10.10 μL), NIPAM (0.25 g), BIS (3.71 mg), and vinyl-modified Fe_3_O_4_ nanoparticles (250 mg) were sequentially added to phosphate buffer (50 mL, 20 mM, pH 5.0). After 15 min purging with N_2_, APS (100 μL, 10%, w/w) and TEMED (10 μL, 5%, w/w) were added to initiate the polymerization. The reaction was allowed to proceed for three hours at 25°C. The particles were then separated using a magnet and washed three times with water. Similarly, to remove the template the particles were eluted with 0.5 M NaCl solution.

*Protein adsorption*: To study the protein adsorption behaviors of the particles, the particles were first dispersed in phosphate buffer (20 mmol, pH 5.0) and incubated at room temperature until reaching swelling equilibrium. After removing the buffer, protein solutions with different initial concentrations were added. After l hour incubation at 25 ^o^C, the mixture was magnetically separated. The protein concentration in the supernatant was determined spectrophotometrically. The adsorption capacity (Q) of the particles were calculated using the following formula:

|  | $Q=\left( C_{0}-C_{e} \right)\times V/W_{d}$ |  |
| --- | --- | --- |

where C_0_ and V represent the initial concentration and volume of the protein solution, respectively. C_e_ represents the remaining protein concentration in supernatant after reaching adsorption equilibrium. W_d_ is the dry weight of particles.

*Separation of BSA from Fetal Bovine Serum*: The particles (25 mg) were dispersed in phosphate buffer (20 mM, pH 5.0) and allowed to swell to equilibrium. After removing the buffer, 10 mL of fetal bovine serum (50-fold diluted with pH 5.0 20 mM phosphate buffer) was added. The mixture was incubated at 25 ^o^C for 1 hour. The particles were separated using a magnet. The adsorbed BSA was then eluted using a 20 mM pH 7.4 phosphate buffer containing 154 mM NaCl. The serum samples before and after adsorption and the eluate from the particles were analyzed using sodium dodecyl sulfate polyacrylamide gel electrophoresis (SDS-PAGE) and high-performance liquid chromatography (HPLC).

*Other Characterizations*: The microscopic morphology of the particles was observed on a JEM-F200 transmission electron microscope. The hydrodynamic diameter (*D_h_*) was measured by dynamic light scattering on a Malvern Zetasizer. Fourier transform infrared spectra (FT-IR) were measured on a Thermo Fisher Nicolet iS20. UV−vis spectra were measured on a Hitachi UV-2910 spectrophotometer. Circular dichroism (CD) spectra were measured at room temperature on a Chirascan V100 spectrometer (Applied Photophysics) using a 0.5 mm quartz cuvette. Sodium dodecyl sulfate polyacrylamide gel electrophoresis (SDS-PAGE) analysis was conducted on a Mini-PROTEAN Tetra electrophoresis system at a constant current of 18 mA. Magnetic properties were measured on a Quantum Design SQUID VSM vibrating sample magnetometer. Thermogravimetric analyses (TG) were conducted using a NETZSCH STA 499 F3 thermogravimetric analyzer. The tests were performed in an oxygen atmosphere using a temperature range of 35-900°C and a heating rate of 10°C per minute. High-performance liquid chromatography (HPLC) analysis was performed on a Shimadzu LC-20ADXR system equipped with a C4 column (4.6 × 250 mm, 5 μm). Mobile phase A was 0.1% trifluoroacetic acid in ultrapure water and mobile phase B was 0.1% trifluoroacetic acid in acetonitrile/water (V/V, 90:10). A linear gradient increase of mobile phase B within 45 min was used with a flow rate of 1.0 mL min^−1^. All samples were detected using a UV detector at 280 nm.

*Statistical Analysis*: All experiments were performed in triplicate. The results are presented as mean ± standard deviation (SD).


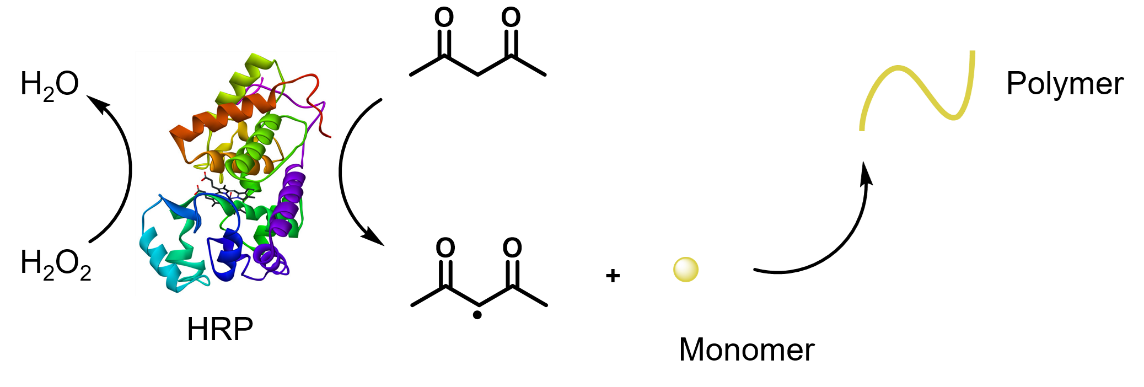


**Scheme S1**. HRP-initiated polymerization.


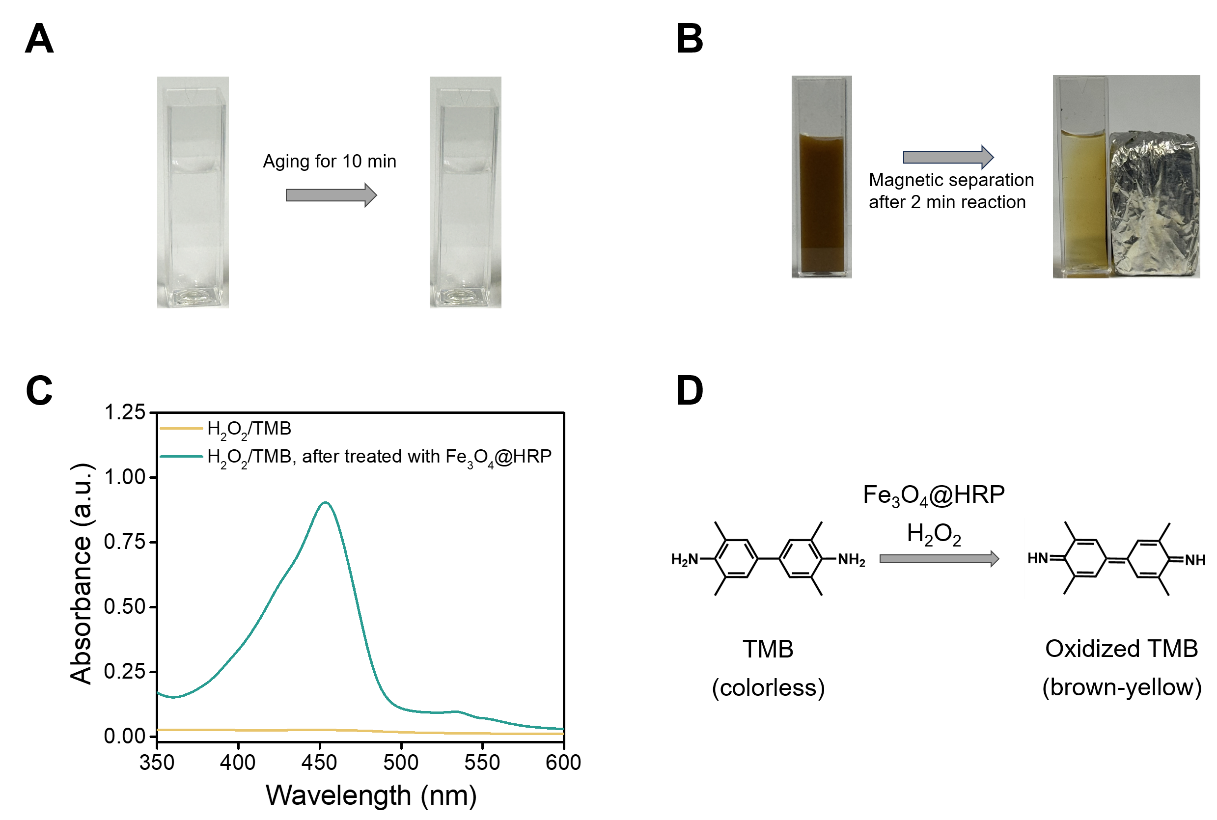


**Figure S1.** (A) Photographs of a H_2_O_2_/TMB mixed solution before and after aging for 10 min. No obvious color change was found. (B) Fe_3_O_4_@HRP NPs were added into a H_2_O_2_/TMB mixed solution. The particles were separated using a magnet after 2 min reaction. The color change indicates the NPs was modified with HRP and the immobilized HRP was catalytically active. (C) UV-vis spectra of a H_2_O_2_/TMB mixed solution and a H_2_O_2_/TMB mixed solution after 2 min reaction with Fe_3_O_4_@HRP NPs. (D) Oxidation of TMB by H_2_O_2_ under catalysis of Fe_3_O_4_@HRP.


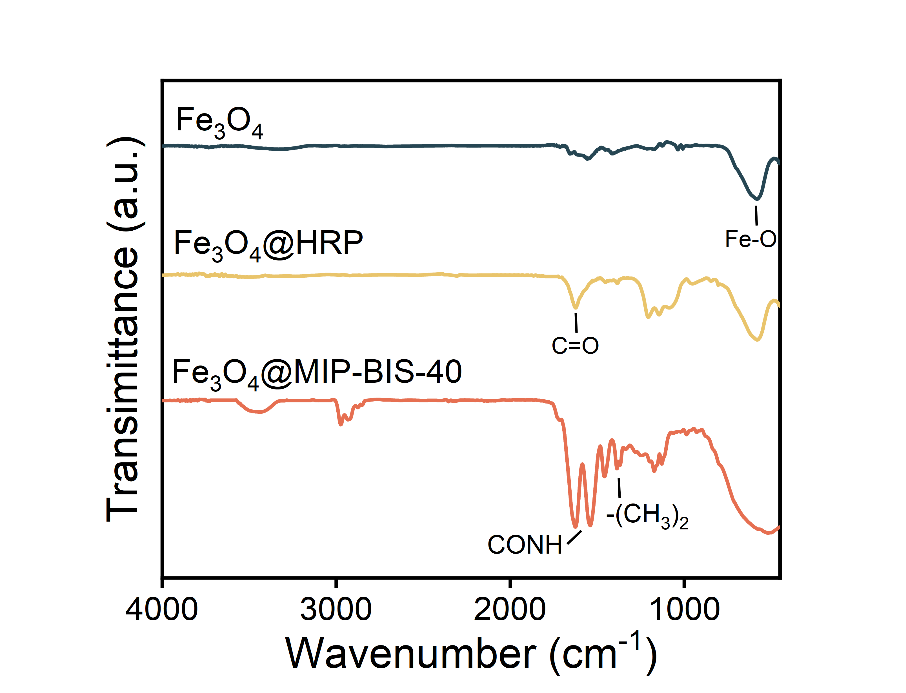


**Figure S2.** FTIR spectra of Fe_3_O_4_, Fe_3_O_4_@HRP and Fe_3_O_4_ @MIP-BIS-40.


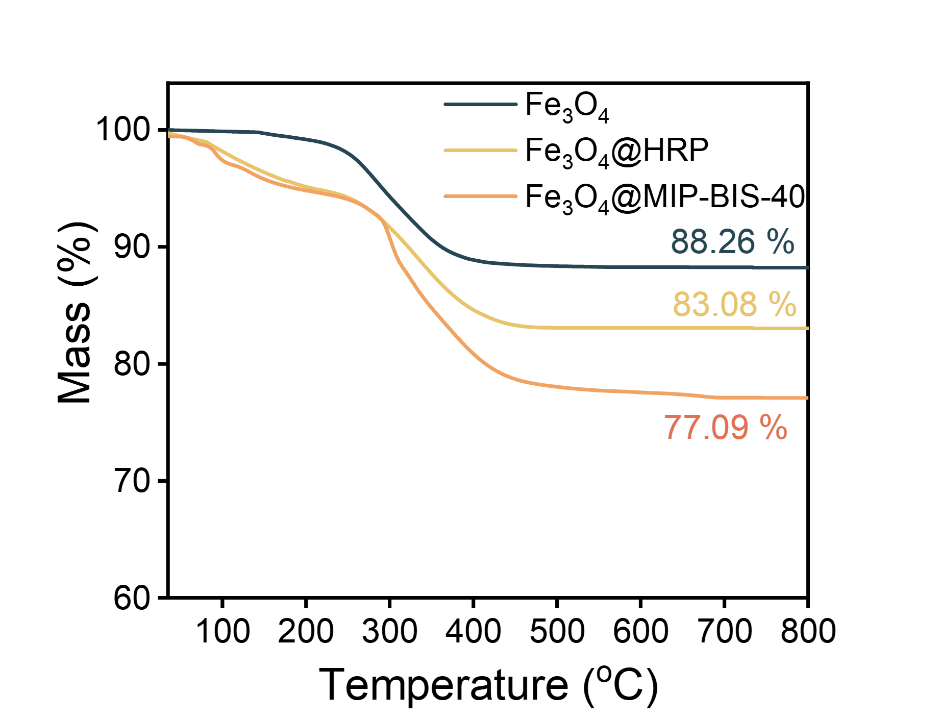


**Figure S3**. TGA curves of Fe_3_O_4_, Fe_3_O_4_@HRP and Fe_3_O_4_ @MIP-BIS-40.


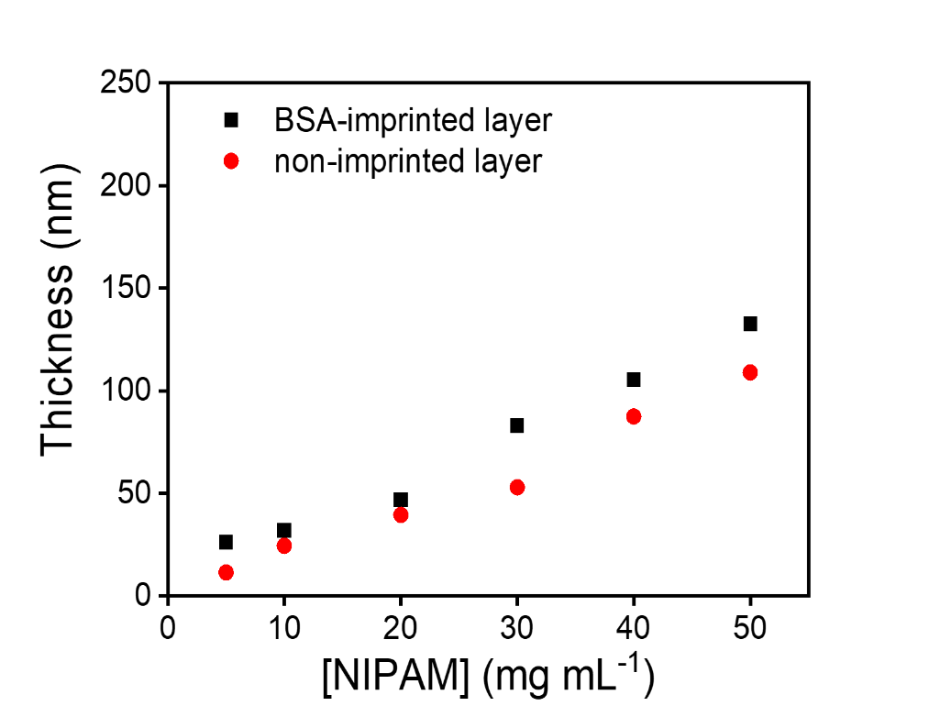


**Figure S4**. Thickness of the BIS-crosslinked BSA-imprinted layer and non-imprinted layer synthesized at different [NIPAM].


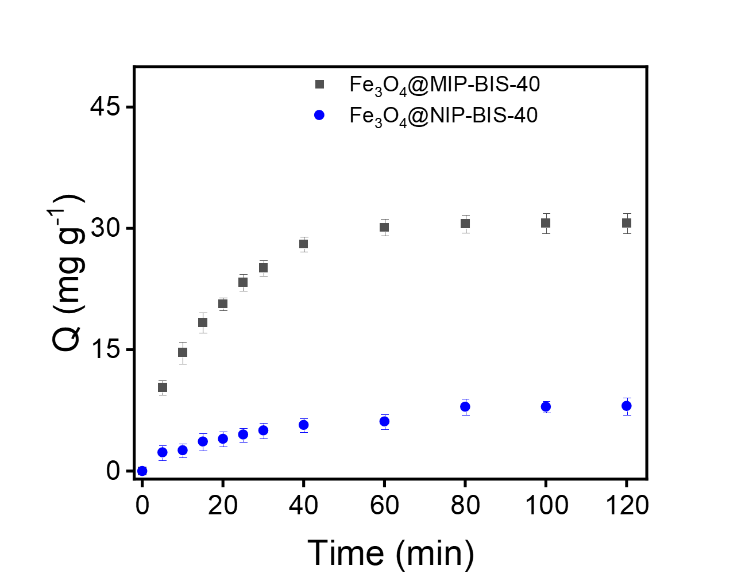


**Figure S5.** Adsorption kinetics of BSA onto the particles. (C_0_ = 0.5 mg/mL, pH = 5.0).


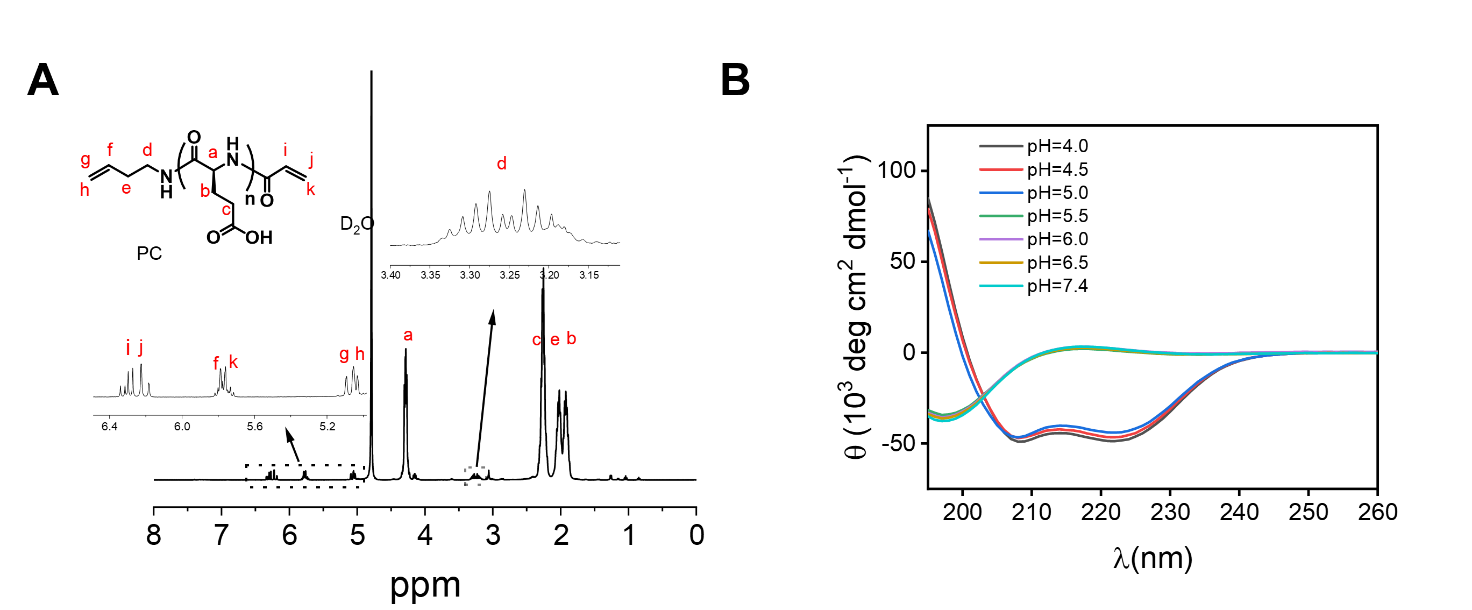


**Figure S6.** (A) ^1^H NMR spectra of the polyglutamic acid-based peptide crosslinker (PC). (B) CD spectra of the PC at different pH.


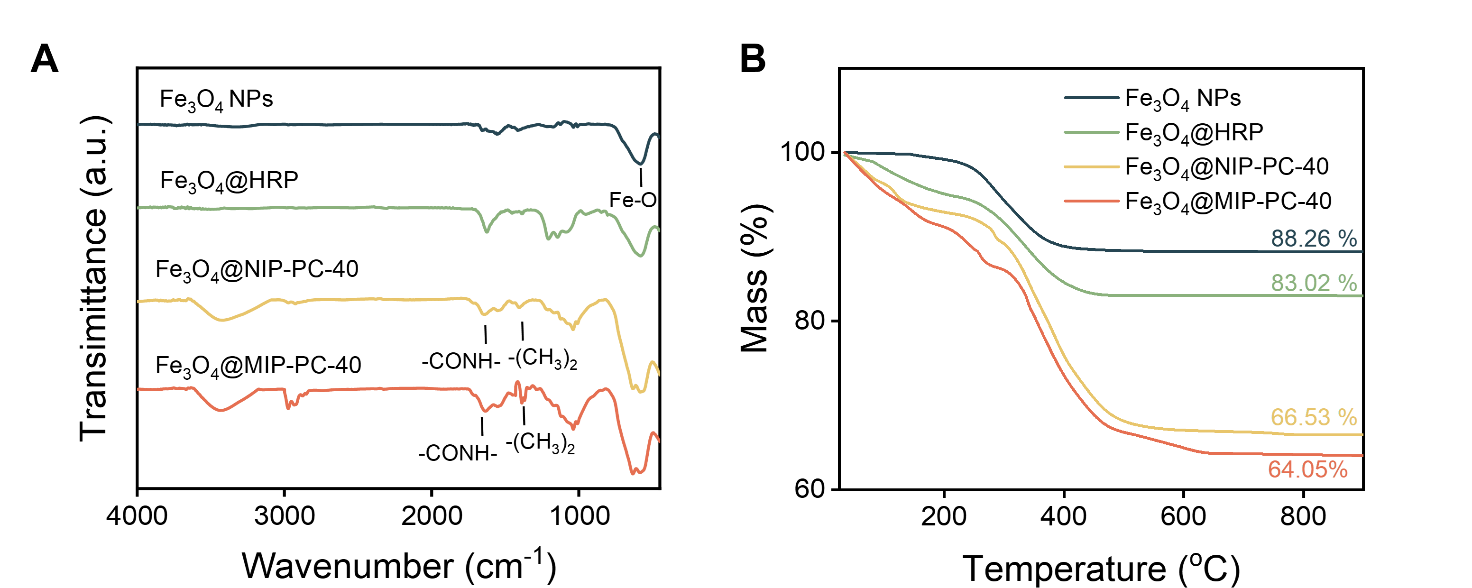


**Figure S7.** FTIR spectra (A) and TGA curves (B) of Fe_3_O_4_, Fe_3_O_4_@HRP, Fe_3_O_4_ @NIP-PC-40 and Fe_3_O_4_ @MIP-PC-40.


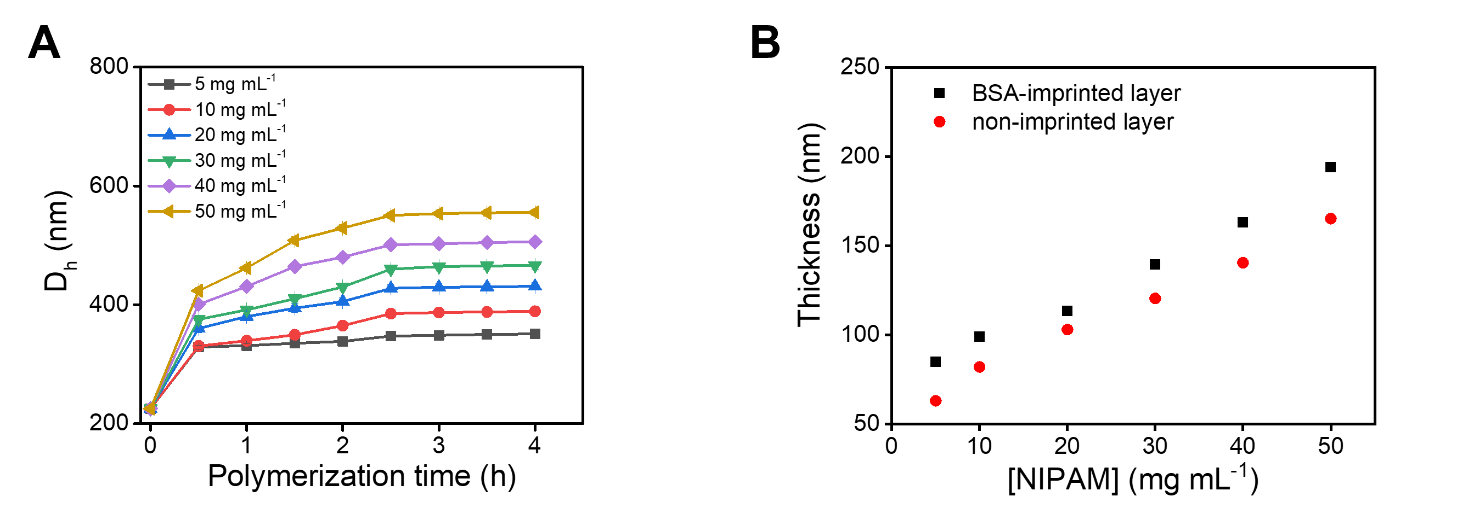


**Figure S8.** (A) D*_h_* of the PC-crosslinked BSA-imprinted particles synthesized using different [NIPAM] and polymerization time. (B) Thickness of the PC-crosslinked BSA-imprinted layer and non-imprinted layer synthesized at different [NIPAM].


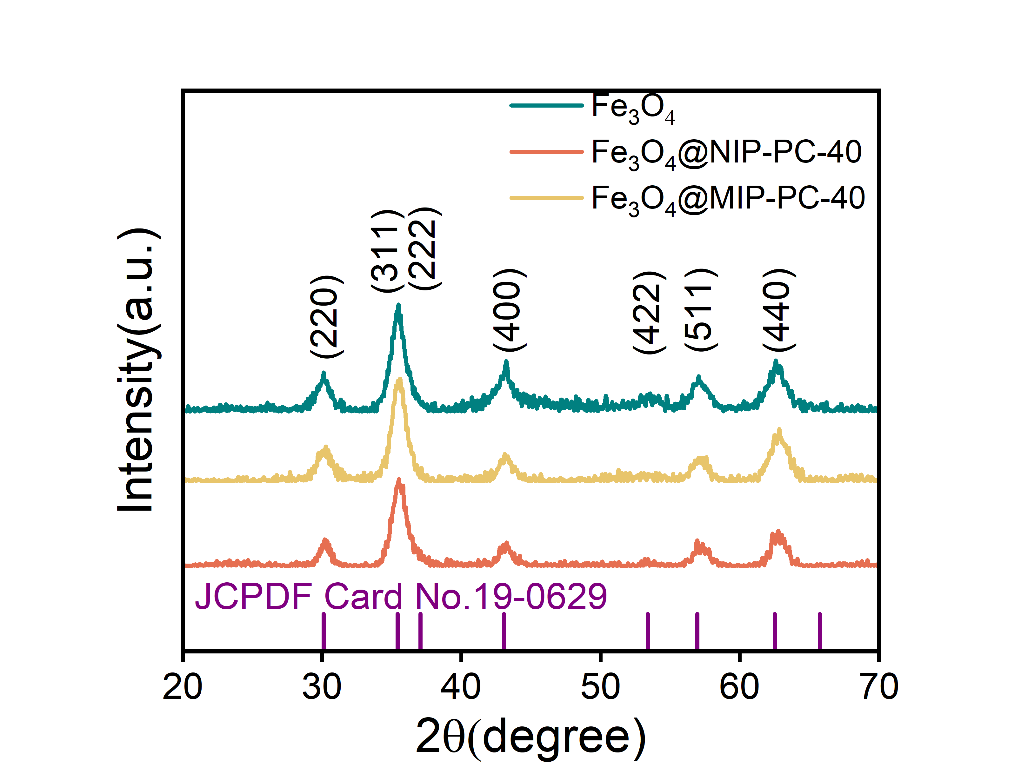


**Figure S9.** XRD patterns of Fe_3_O_4_, Fe_3_O_4_@NIP-PC-40 and Fe_3_O_4_@MIP-PC-40.

**Table S1**. Composition of the pre-polymerization solutions for the BIS-crosslinked imprinted and non-imprinted Fe_3_O_4_ NPs. The monomers and crosslinker were dissolved in 50 mL of 20 mM pH 5.0 phosphate buffer.

| Samples | [NIPAM]  (mg mL^-1^) | NIPAM  (g) | AAM  (mg) | MAA  (μL) | DMAEMA  (μL) | BIS  (mg) | BSA  (mg) |
| --- | --- | --- | --- | --- | --- | --- | --- |
| Fe_3_O_4_@NIP-BIS-5 | 5 | 0.25 | 5.70 | 5.10 | 10.10 | 3.71 | 0 |
| Fe_3_O_4_@NIP-BIS-10 | 10 | 0.50 | 11.31 | 10.11 | 20.10 | 7.42 | 0 |
| Fe_3_O_4_@NIP-BIS-20 | 20 | 1.00 | 22.61 | 20.23 | 40.20 | 14.84 | 0 |
| Fe_3_O_4_@NIP-BIS-30 | 30 | 1.50 | 33.92 | 30.34 | 60.31 | 22.26 | 0 |
| Fe_3_O_4_@NIP-BIS-40 | 40 | 2.00 | 45.22 | 40.45 | 80.41 | 29.68 | 0 |
| Fe_3_O_4_@NIP-BIS-50 | 50 | 2.50 | 56.53 | 50.09 | 100.51 | 37.09 | 0 |
| Fe_3_O_4_@MIP-BIS-5 | 5 | 0.25 | 5.70 | 5.10 | 10.10 | 3.71 | 40.00 |
| Fe_3_O_4_@MIP-BIS-10 | 10 | 0.50 | 11.31 | 10.11 | 20.10 | 7.42 | 40.00 |
| Fe_3_O_4_@MIP-BIS-20 | 20 | 1.00 | 22.61 | 20.23 | 40.20 | 14.84 | 40.00 |
| Fe_3_O_4_@MIP-BIS-30 | 30 | 1.50 | 33.92 | 30.34 | 60.31 | 22.26 | 40.00 |
| Fe_3_O_4_@MIP-BIS-40 | 40 | 2.00 | 45.22 | 40.45 | 80.41 | 29.68 | 40.00 |
| Fe_3_O_4_@MIP-BIS-50 | 50 | 2.50 | 56.53 | 50.09 | 100.51 | 37.09 | 40.00 |

**Table S2**. Composition of the pre-polymerization solutions for the PC-crosslinked imprinted and non-imprinted Fe_3_O_4_ NPs. The monomers and crosslinker were dissolved in 50 mL of 20 mM pH 5.0 phosphate buffer.

| Samples | [NIPAM]  (mg mL^-1^) | NIPAM  (g) | AAM  (mg) | MAA  (μL) | DMAEMA  (μL) | PC  (mg) | BSA  (mg) |
| --- | --- | --- | --- | --- | --- | --- | --- |
| Fe_3_O_4_@NIP-PC-5 | 5 | 0.25 | 5.70 | 5.10 | 10.10 | 68.22 | 0 |
| Fe_3_O_4_@NIP- PC -10 | 10 | 0.50 | 11.31 | 10.11 | 20.10 | 136.44 | 0 |
| Fe_3_O_4_@NIP- PC -20 | 20 | 1.00 | 22.61 | 20.23 | 40.20 | 272.88 | 0 |
| Fe_3_O_4_@NIP- PC -30 | 30 | 1.50 | 33.92 | 30.34 | 60.31 | 409.32 | 0 |
| Fe_3_O_4_@NIP- PC -40 | 40 | 2.00 | 45.22 | 40.45 | 80.41 | 545.76 | 0 |
| Fe_3_O_4_@NIP- PC -50 | 50 | 2.50 | 56.53 | 50.09 | 100.51 | 682.20 | 0 |
| Fe_3_O_4_@MIP- PC -5 | 5 | 0.25 | 5.70 | 5.10 | 10.10 | 68.22 | 40.00 |
| Fe_3_O_4_@MIP-PC-10 | 10 | 0.50 | 11.31 | 10.11 | 20.10 | 136.44 | 40.00 |
| Fe_3_O_4_@MIP-PC-20 | 20 | 1.00 | 22.61 | 20.23 | 40.20 | 272.88 | 40.00 |
| Fe_3_O_4_@MIP-PC-30 | 30 | 1.50 | 33.92 | 30.34 | 60.31 | 409.32 | 40.00 |
| Fe_3_O_4_@MIP-PC-40 | 40 | 2.00 | 45.22 | 40.45 | 80.41 | 545.76 | 40.00 |
| Fe_3_O_4_@MIP-PC-50 | 50 | 2.50 | 56.53 | 50.09 | 100.51 | 682.20 | 40.00 |

References

[1] R. Liu, H. Wang, W. Lu, L. Cui, S. Wang, Y. Wang, Q. Chen, Y. Guan, Y. Zhang, *Chem. Eng. J.* **2021**, *415*, 128839.

[2] Y. Zhang, Y. Wang, Y. Guan, Y. Zhang, *Nat. Commun.* **2022**, *13*, 6671.

[3] Y. Wang, Y. Zhang, Y. Guan, Y. Zhang, *Langmuir* **2022**, *38*, 6057.

[4] H. He, G. Fu, Y. Wang, Z. Chai, Y. Jiang, Z. Chen, *Biosen. Bioelectron.* **2010**, *26*, 760.
